# Minoritised ethnic groups and modifiable dementia risk: a scoping review of UK-based evidence

**DOI:** 10.1136/jech-2024-222654

**Published:** 2025-04-17

**Authors:** Magda Jordão, Lin Gong, Deirdre Andre, Amirah Akhtar, Emmanuel Nwofe, Rebecca Hawkins, Kate Best, Sahdia Parveen, Karen Windle, Andrew Clegg

**Affiliations:** 1 Academic Unit for Ageing and Stroke Research, University of Leeds, Leeds, UK; 2 Academic Unit for Ageing and Stroke Research, Bradford Institute for Health Research, Bradford Teaching Hospitals NHS Foundation Trust, Bradford, UK; 3 University of Leeds Libraries, University of Leeds, Leeds, UK; 4 Centre for Applied Dementia Studies, University of Bradford, Bradford, UK

**Keywords:** DEMENTIA, ETHNIC GROUPS, Health inequalities, PREVENTION, PUBLIC HEALTH

## Abstract

**ABSTRACT:**

**Background:**

People from minoritised ethnic groups are more likely to be impacted by dementia. In the general population, dementia may be prevented or delayed by up to 40% by reducing risk in 12 modifiable risk factors (MRF). However, minoritised ethnic groups are not systematically included.

**Objectives:**

We conducted a scoping review following Joanna Briggs Institute guidance to map: (1) which minoritised ethnic groups have been included in UK research on dementia MRF, (2) for which MRF and (3) using which research methods.

**Eligibility criteria:**

Eligible studies analysed one or more of the 12 MRFs among minoritised ethnic groups.

**Evidence sources:**

Medline, Embase Classic+Embase, PsycInfo, Web of Science, CINAHL and grey literature were searched.

**Charting methods:**

Patient and public involvement with minoritised ethnic groups and professionals informed the data extraction tool. We use frequencies and graphs in data description.

**Results:**

We screened 7748 records, assessed 122 full text records and included 14 studies, which mostly used broad ethnic groups. Hypertension, diabetes and depression were studied as predictors of dementia in 10, eight and six studies, respectively, compared with low social contact and air pollution in just two each. Measures of MRF lacked consistency, and data per ethnic group were not reported in several studies. Research examining interactions in combinations of MRFs was lacking.

**Conclusions:**

More research is needed with specific ethnic groups, consistent measures and focusing on discrimination and MRF interaction and severity. This will be key to personalised risk reduction with diverse communities.

**WHAT IS ALREADY KNOWN ON THIS TOPIC**

**WHAT THIS STUDY ADDS**

- For the first time, we used evidence synthesis methods and patient and public involvement to map UK evidence gaps.
- There was a lack of research for many of the modifiable risk factors, and the ethnic groups included were broad.
- The interaction between risk factors was one of the most important aspects to patient and public contributors, but there was almost no research available on this.

**HOW THIS STUDY MIGHT AFFECT RESEARCH, PRACTICE OR POLICY**

- A greater research focus on fine-grained ethnicity groups and on the interplay between risk factors can contribute to support better intervention prioritisation and personalised risk reduction with minoritised ethnic groups.

## Introduction

 Dementia is one of the greatest global health challenges,^1^ coming at high societal cost, reducing life expectancy and increasing disability.^2 3^ Characterised by reduced cognitive ability and compromised everyday function,^2^ dementia affects almost 1 million people in the UK and is expected to keep rising to 1.7 million in England and Wales by 2040.^4^

The prevalence of dementia could be reduced by 40% through prevention of 12 key modifiable risk factors (MRF), based on a life course model.^5 6^ These include education, hypertension, hearing impairment, traumatic brain injury (TBI), obesity, alcohol consumption, depression, physical inactivity, diabetes, low social contact, smoking and air pollution.

Some minoritised ethnic groups have a higher incidence of dementia.^7^ Following the WHO framework, these groups are defined by self-identifying as sharing an ethnicity (eg, based on language, culture), being smaller in number than the rest of the population and not having a dominant position socially, economically or politically.^8^ Ethnic categories are often defined widely, against recommendations, and consensus on categorisation is an ongoing endeavour.^9^ In England and Wales, the majority of the population is white British (74.4%), and other ethnic groups are minoritised ethnic groups, with Indian (3.1%), Pakistani (2.7%) and black African (2.5%) among the most common, all of which have been increasing in median age.^10 11^

The incidence of dementia is higher among people of black ethnicity,^12^ and prevalence is expected to rise more rapidly for minoritised ethnic groups compared with white British.^13^ Furthermore, people of African Caribbean ethnicity are more likely to experience vascular dementia,^14^ while people from South Asian and African Caribbean ethnicities are more likely to experience dementia earlier than white British.^14 15^

Ethnic inequalities in dementia incidence may be partially driven by higher prevalence of MRFs. For example, in the UK, type II diabetes is more prevalent in black and South Asian adults than in white adults.^16^ The level of dementia risk associated with each MRF may also differ by ethnicity, but this has not been thoroughly investigated, with dementia prevention research failing to systematically involve people from minoritised ethnic groups.^17^

Scoping reviews are useful for identifying research gaps and guiding future research. We aimed to assess the scope and research gaps on the 12 MRFs with minoritised ethnic groups in the UK using scoping review methods. We aimed to investigate: (1) which minoritised ethnic groups have been studied, (2) for which of the 12 MRFs and (3) using which methods.

## Methods

We followed the Joanna Briggs Institute (JBI) guidance^18^ and the Preferred Reporting Items for Systematic Reviews and Meta-Analyses extension for Scoping Reviews (PRISMA-ScR).^19^ This scoping review was registered,^20^ and the PRISMA-ScR checklist is provided (online supplemental file 1). Patient and public involvement (PPI) and knowledge user engagement activities were based on JBI guidance.^21^

### Eligibility criteria

As per the JBI guidance, the eligibility criteria focused on participants, concept, and context and the type of evidence sources.

#### Participants

Studies had to include and analyse at least a subset of the participants from a minoritised ethnic group (non-white British).

#### Concept

Studies had to focus on the association of one or more of the 12 MRFs with a dementia diagnosis and/or cognitive impairment related to dementia.^2^ This could include studies testing interventions on the 12 MRFs to change dementia diagnosis and/or cognition.

The association could either be reported in the individual minoritised ethnic group (eg, the relative risk of dementia associated with hypertension in South Asian adults only), or in a group of several ethnicities, if the interaction between the dementia MRF and ethnicity was included.

We excluded studies focusing exclusively on other risk factors (eg, genetics), not analysing the impact on dementia diagnosis and/or cognition, and analysing dementia diagnosis and/or cognition and the 12 MRFs as covariates only.

#### Context

We initially planned to include studies in any context. However, discussions with PPI contributors (see the Patient and public involvement and knowledge user engagement section) emphasised the need to consider context specificities. Accordingly, we included only UK studies, implementing this at the study selection stage. This is in line with literature recognising that cross-country comparisons in this topic are very complex and less relevant due to differences in migration history, politics and services.^22^

When the location was not explicitly mentioned, non-UK context was inferred based on (a) ethnic minority categories not used in the UK (eg, African-American, Latino, Hawaiian), (b) participants from non-UK cohort, (c) funding by a non-UK governmental organisation and (d) all authors affiliated with non-UK institution.

#### Types of sources

We included studies reporting empirical evidence on a quantitative measure of risk or its systematic synthesis. Grey literature sources (eg, conference abstracts, unpublished theses) were eligible. Exclusions included narrative reviews, qualitative studies, protocols, commentaries and editorials, which do not provide evidence on risk.

### Search strategy

The search strategy (online supplemental file 2) was developed by an information specialist (DA) and discussed with the team. Relevant subject headings and free text words were identified. The search strategy was piloted in Medline, contrasted with studies of interest and adjusted (eg, adding terms). This was peer reviewed and translated across databases. We searched: Medline (ALL), Embase Classic+Embase, APA PsycInfo, CINAHL and Web of Science without language or date restrictions. The final search was performed on 21 May 2023.

We searched the grey literature, including conference proceedings, organisational and thesis databases. The grey literature search development was similar to the database search but constrained to UK-based studies. This was completed on 22 November 2023. Checks on reference lists of related reviews were conducted as well as citation searching.

### Study selection

Records were managed using EndNote^23^ and Covidence.^24^ Title and abstract screening was piloted for 30 records. Reviewers (MJ, AA, LG) conducted independent double screening in 5.3% of the 7713 ‘title and abstract’ records identified in the database search. At this point, analysis of the inter-reviewer agreement revealed substantial to perfect agreement (Cohen’s kappa=0.78–0.84), and the screening continued with a single reviewer. Conflicts were resolved through discussion between reviewers and the team. Full text papers and the grey literature were double screened for 10% of the records with perfect agreement (100%), followed by single screening (MJ). There were two records for which it was not possible to access full text, but these were considered unlikely to have been relevant (one Japanese report and one poster abstract in which the study was not completed).

### Data extraction

Data extraction was conducted using Covidence^24^ and piloted for 10% of studies by two independent reviewers. After piloting, one reviewer (MJ) continued the data extraction consulting the team as necessary (see online supplemental file 3 for extraction form).

### Data analysis and synthesis

The extracted data were tabulated. Some fields for which verbatim data were extracted were organised into categories by MJ (ethnicity and risk factor measures, and outcomes).

We generated percentages and frequencies to support narrative descriptions and graphical representations.

### Patient and public involvement and knowledge user engagement

Following the JBI guidance,^21^ we developed PPI and knowledge user engagement (methodology in ref 20). We involved six people from minoritised ethnic communities with various ages and education levels, and four professionals with experience in dementia across primary, secondary and social care.

We discussed gaps and priorities in research. Contributors highlighted the importance of (1) context, (2) the interaction between MRFs and (3) additional factors, namely: racism, discrimination, deprivation, low income, housing, sound pollution, diet, sleep, visual impairment and olfactory (smell) impairment.

## Results

**Figure 1:**
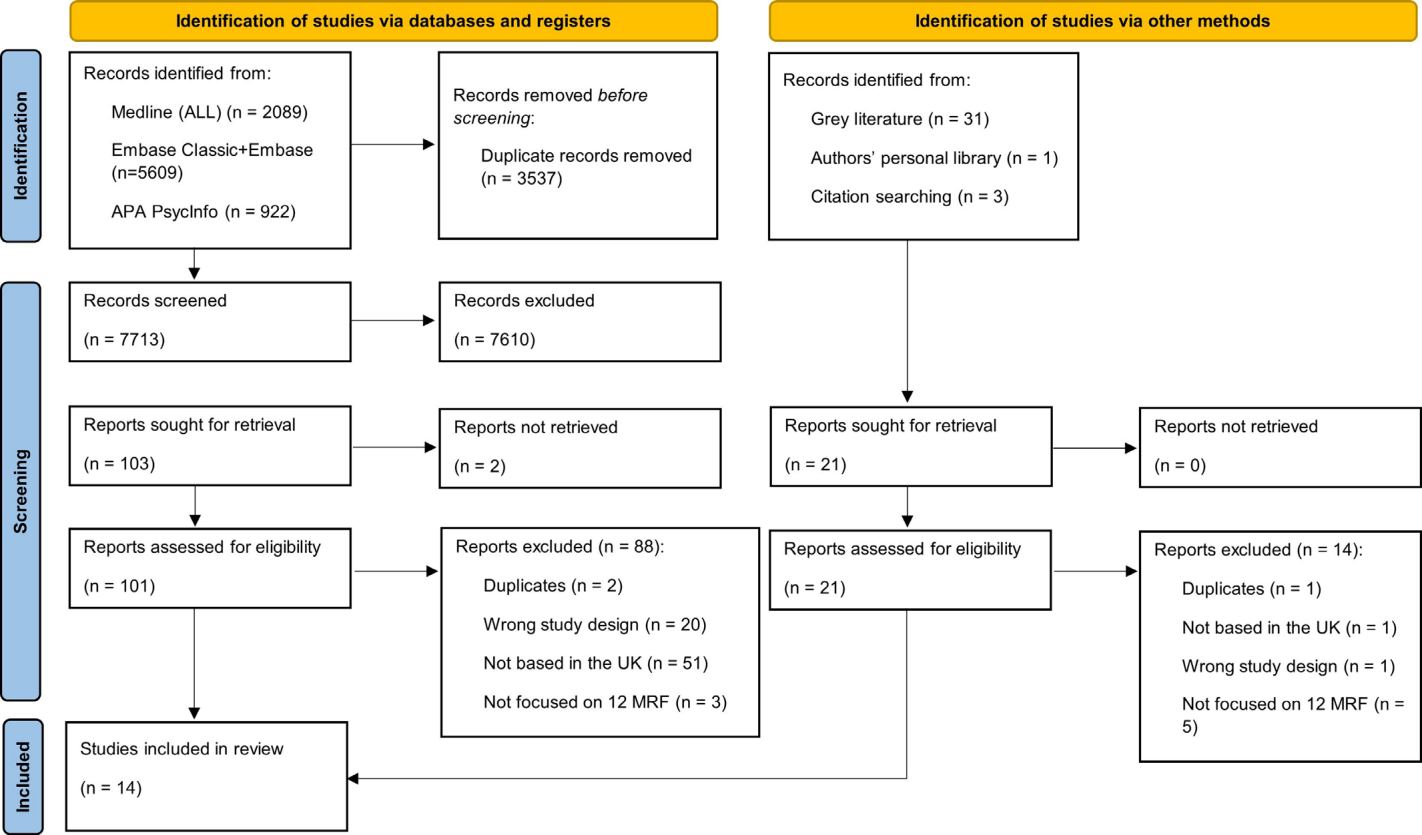
Preferred Reporting Items for Systematic Reviews and Meta-Analyses (PRISMA) diagram (the number of studies included is lower than the reports due to some studies being described in more than one report, as identified in the Study ID column, online supplemental file 4). MRF, modifiable risk factors.

The search results are detailed in the PRISMA diagram (figure 1).

### Study characteristics

Study characteristics are described in online supplemental file 4. One of the studies^25^ was a systematic review summarising studies already included. This review is described but not included in the results to avoid duplication.

Overall, seven studies were published since 2018. In contrast, six studies were published in a span of 17 years (2000–2017).

### Which methods have been used?

#### Study design, data source and recruitment

The most frequent study design was cohort study (6), followed by cross-sectional (4) and case–control studies (3). Secondary data using medical records included five studies, and cohort data in one study. Primary data involving new data collection were used in seven studies. Most studies recruited from England (10), with two studies in England, Scotland and Wales, and one study in all UK nations. Nine studies took place in a single specific location, eight of which in London (see online supplemental file 4 for sampling details).

#### Outcomes

Most studies used a dementia diagnosis on its own (7, 54%) or alongside a cognitive assessment (2, 15%). The diagnosis was ascertained from medical records (5, 38%), medical records or self-report (2, 15%), or was a clinical diagnosis based on a cognitive assessment conducted as part of the study (2, 15%). Four studies used cognitive assessment only (31%). The cut-off to define cognitive impairment was not externally validated in any study.

#### Data analysis

Most studies adjusted the analyses. Of the nine studies including an analysis of MRF by minoritised ethnic group, five were adjusted, two unadjusted and two presented both analyses. For the seven studies presenting the interaction between 12 MRFs and ethnicity, six were adjusted, and only one unadjusted.

### Which minoritised ethnic groups have been studied?

**Figure 2:**
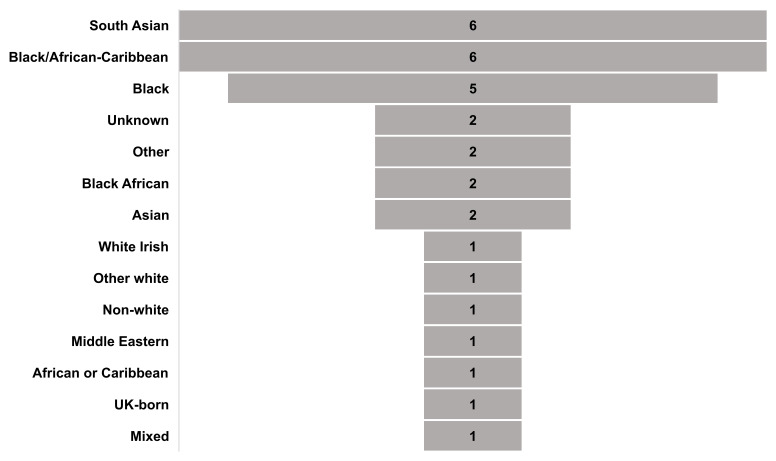
Number of studies per minoritised ethnic groups sampled. Ethnic group names replicate those used in included studies.

As represented in figure 2, the ethnic groups studied were often broad (eg, South Asian, black), reinforcing concerns with homogenisation.^26^ In some studies, more specific categories were included in the sample but not analysed separately (online supplemental file 5).

The ‘other white’ category was under-represented compared with the ethnic breakdown in England and Wales in the 2021 Census.^11^

#### Ethnicity measures

The most common ethnicity measure sources were medical records and self-reported ethnicity based on UK census categories in four studies each (31%). In two studies (15%), ethnicity was attributed by researchers based on appearance and parental origin, and in the other two (15%), it was initially determined by the staff and subsequently confirmed by participants’ self-report. One study (8%) used self-reported country of birth.

### Which of the 12 MRFs have been studied?

**Figure 3:**
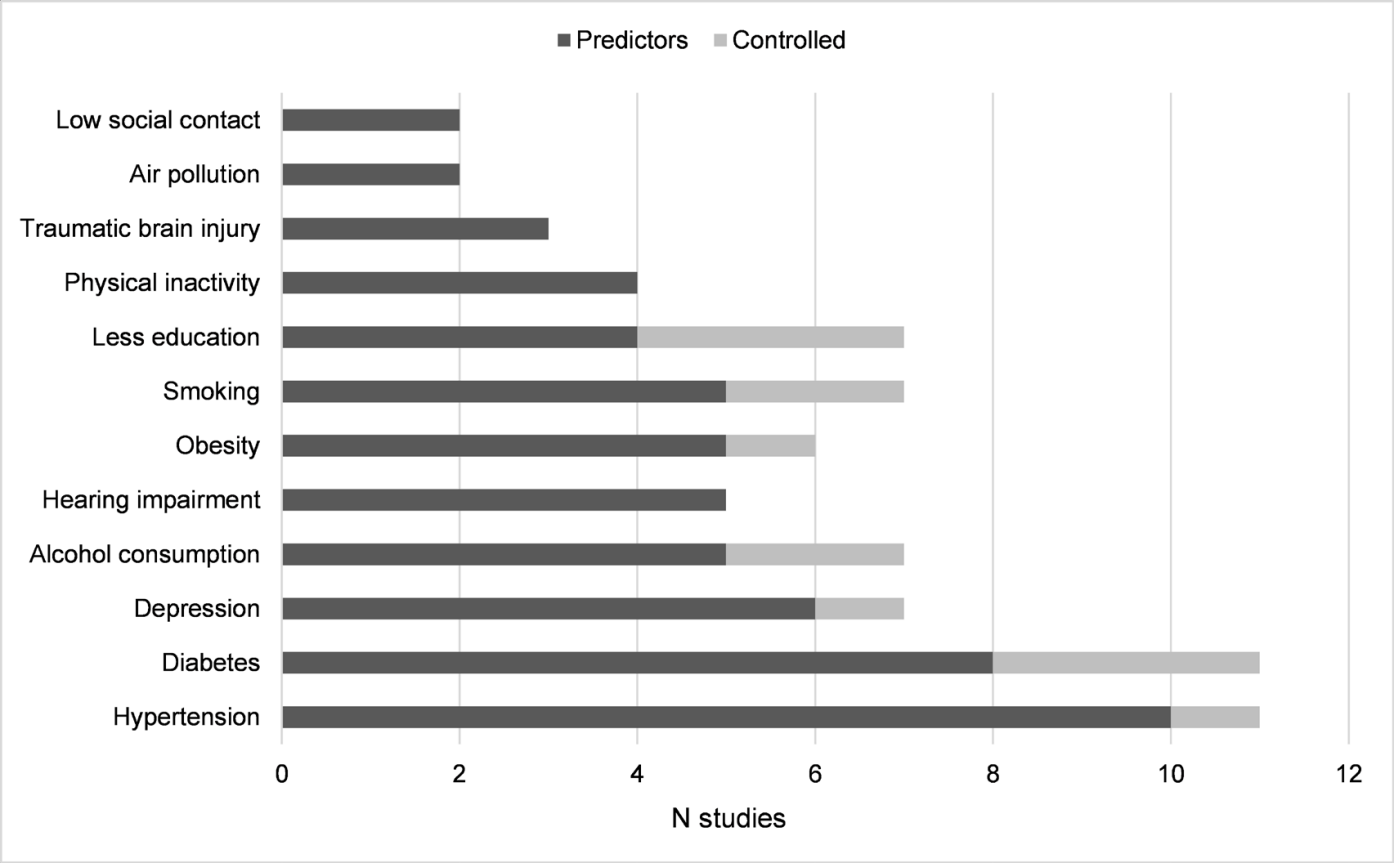
Number of studies per 12 modifiable risk factors (MRF).

Current literature with minoritised ethnic groups is limited, especially for the MRF which may have the greatest potential for prevention in the general population. Hypertension and diabetes, the two most commonly studied MRFs (figure 3), have been found to have the potential to contribute to only 3% of dementia cases in the general population.^6^ In contrast, low social contact, one of the least studied risk factors in minoritised ethnic groups, may have a 4% contribution.^6^

#### Twelve MRF measures

Risk factors were measured based on various sources, namely physical examination, medical record and/or self-report. Operational definitions of MRFs varied; for example, ‘less education’ was defined in different studies as less than 10 years of education and leaving school with less than 15 years old. The MRFs were rarely analysed as continuous variables (eg, diabetes as present/absent rather than glucose readings).

#### Additional risk factors

Four of the risk factors mentioned by PPI were studied: olfactory impairment (1), housing (1), sleep (1) and socioeconomic deprivation (4). The impact of racism and discrimination, visual impairment, low income, sound pollution and diet were not included in any of the studies.

#### Interaction between 12 MRFs

We identified only two studies analysing the interaction between risk factors. One of these analysed the interaction of education with diabetes, hypertension and physical inactivity.^27^ The other study analysed the interaction of deprivation with hypertension, hearing impairment, smoking, obesity, depression, diabetes, alcohol consumption and TBI.^15^

### Which of the minoritised ethnic groups have been studied for which MRF?

We investigated how minoritised ethnic groups and MRF intersected (online supplemental file 5). Some groups were described as part of the sample but not analysed as a subgroup for the MRF of interest. When MRFs were included as covariates, there were often also no data per subgroup.

The interaction between ethnicity and MRF (instead of in addition to presenting a subgroup analysis) was presented in seven studies.

## Discussion

**Figure 4:**
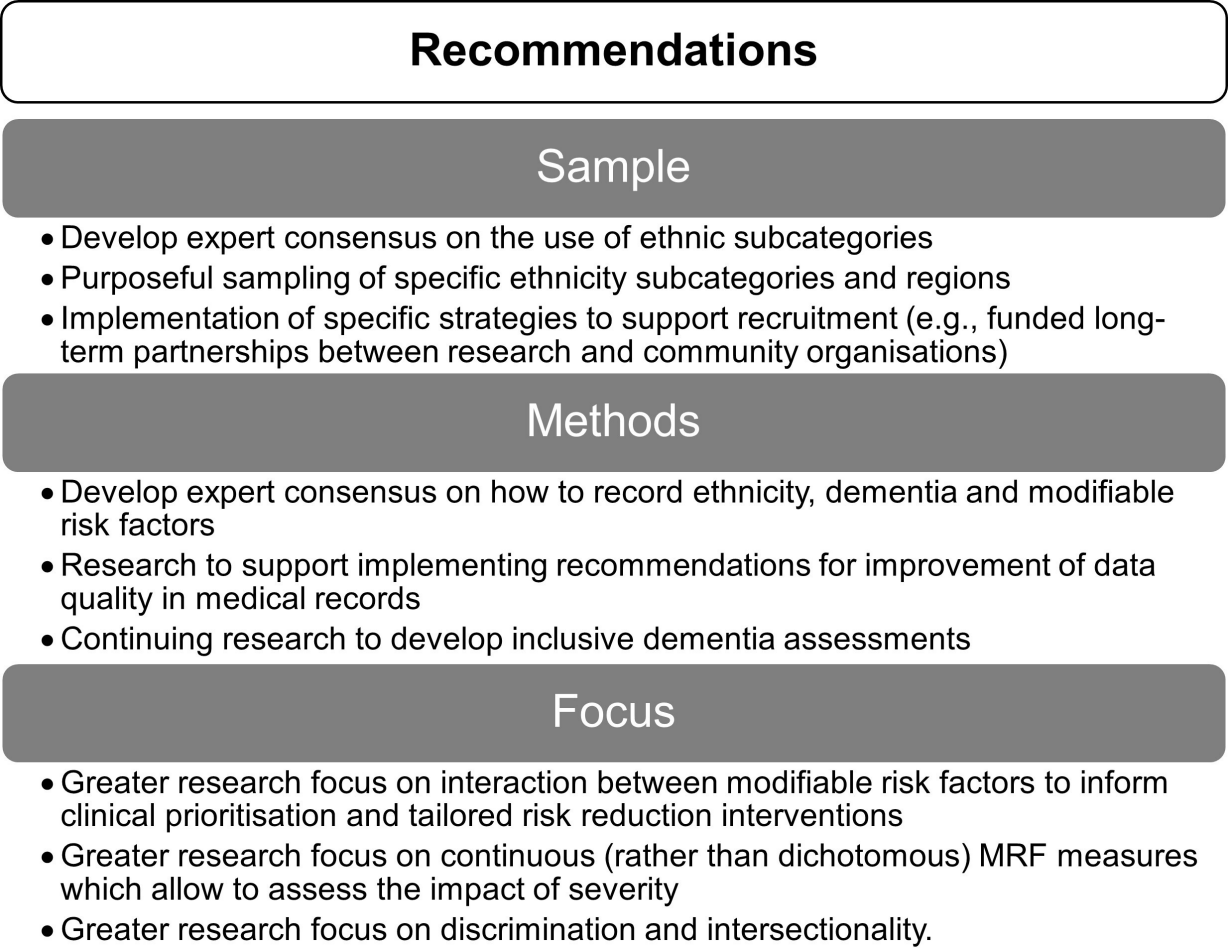
Summary of key recommendations. MRF, modifiable risk factors.

This review provides the first research mapping on the 12 dementia MRFs among minoritised ethnic groups in the UK, clarifying research gaps and priorities for future research. We found increasing publications in recent years, suggesting a growing recognition of the importance of this area. We identified key gaps and recommendations for sample, methods and research focus (figure 4).

### Sample

#### Gaps

Ethnicity often focused on high-level categories from the 2021 Census, improving consistency across studies, but there was still variation in the categories used. Overall, analyses of ethnicity subcategories were lacking. In medical records, a common data source, 16 ethnic groups are specified,^28^ but these were grouped for analysis, risking homogenisation. In some primary studies, specific ethnic groups are described as part of the sample but not detailed in analysis (online supplemental file 5).

Another relevant aspect of sampling is the region of recruitment. Most studies were based in London, with no studies focusing specifically on other multicultural areas, such as Bradford.

#### Recommendations

Future research needs to intentionally improve the recruitment of specific minoritised ethnic groups to avoid homogenisation.^26^ Improved recruitment is particularly important in cohort studies, including cohorts specifically focused on minoritised ethnic groups, which have been key to this research area in the USA (eg, Honolulu-Asia Aging Study), or targeted oversampling of minoritised ethnic groups. This will depend on implementing existing knowledge: the difficulties in diverse recruitment have been mapped,^29^ and recruitment toolkits are available.^30^ In this study, PPI contributors emphasised the need for funded long-term partnerships with community organisations which can bridge researchers and participants.

Recruiting specific groups will require better understanding and guidelines on ethnic categorisation. Recent research suggests that consensus exercises and regular updates are useful.^9^ This could be further developed for the UK context. Meanwhile, we recommend following UK Office for National Statistics ethnicity subcategories as a well-standardised approach.

Further research in multiethnic areas of the UK is also recommended to assess generalisability and better understand the role of context, for example, the interaction with socioeconomic deprivation.

### Methods

#### Gaps

We found an increasing tendency to rely on medical records to determine ethnicity (a third of all studies, and half of those published in the last 5 years). While routine health and administrative data bring advantages, including statistical power to investigate interactions across ethnicity and access to detailed health information, it also presents challenges. Recent reports indicate that the use of specific ethnic groups is not standardised and may be inaccurate.^28^

Using records may also impact the accuracy of dementia diagnosis compared with primary data collection. Relying on recorded dementia diagnoses probably underestimates dementia cases and MRF risk in minoritised ethnic groups due to greater difficulties accessing diagnosis^31^ and lack of dementia awareness in some communities.^32^ Dementia assessments are also not unbiased, as there is a lack of externally validated cognitive assessments with minoritised ethnic groups.^33^

Finally, MRFs were measured based on various sources. This is problematic for aggregating and understanding results across studies.

#### Recommendations

Identifying common definitions and reliable data sources is particularly challenging, but recent research can be expanded to support improvement. Namely, difficulties in collecting ethnicity data in health services have been mapped, and recommendations for improvement have been developed with healthcare workers.^34^ Projects to support the implementation of these recommendations and further consensus work with expert groups on how to record ethnicity and dementia will be key.

Regarding the bias in dementia assessment, efforts to develop adequate tools and training are underway across Europe.^35^ Integrating these in future MRF research will be important.

Discussion and consensus on the preferred measurement of the 12 MRFs among an expert group including researchers, clinicians and PPI contributors are also recommended.

### Focus

#### Gaps

We found only two studies analysing the interaction between the 12 MRFs in minoritised ethnic groups, at odds with the PPI contributors’ focus. The 12 MRFs were rarely measured continuously, limiting the ability to understand the impact of severity. Finally, research was lacking on the impact of racism and discrimination, which was emphasised in PPI discussion.

#### Recommendations

Further research on how risk factors interact is recommended. This is key to understanding combinations of risk factors which not only add risk but interact to amplify it (eg, depression amplifying low social contact and physical inactivity). Some risk factors may also cause others to be present and should be an intervention priority. This research could inform clinical prioritisation and be considered in personalised risk reduction programmes, which are expected to be an essential part of dementia risk reduction services.^36^ Investing in understanding how to tailor risk reduction may also reveal a more effective approach. A network meta-analysis of complex interventions to promote independence in ageing revealed that interventions providing tailored multidomain interventions, rather than providing everything for all, have the best evidence for effectiveness.^37^ Similarly, multidomain interventions providing risk reduction for several risk factors simultaneously and across all participants may not be more effective and entail more costs. Research informing how to tailor interventions is key.

Research on severity will also be important, for example, by analysing risk factors continuously (eg, using blood pressure readings instead of with/without hypertension). If specific levels of risk severity can be identified as problematic, these could be a priority.

More research on discrimination is needed, as it has been shown in other countries that it impacts brain health^38^ and cognitive ageing.^39^ More broadly, considering how structural factors of inequality interact, following an intersectionality perspective, will be important to dementia prevention (as for dementia care).^40^

### Limitations and strengths

Limitations include broad rather than in-depth analysis of the literature, which is inherent to scoping review methods, but allowed for an overview of gaps and priorities. The variability of MRF and ethnicity measures can be considered a limitation to summarising the evidence. Conversely, the study allowed us to highlight these gaps.

Methodologically, we followed a systematic process of searching and data charting and involved PPI contributors. Only part of the records was double screened, which is a limitation, but the substantial to perfect agreement is reassuring.

## Conclusion

Existing research on 12 MRFs among minoritised ethnic groups in the UK is limited and inconsistent. We summarise the recommendations (figure 4) which could support personalised risk reduction strategies and avoid further inequities.

## Supplementary material

10.1136/jech-2024-222654 online supplemental file 1

10.1136/jech-2024-222654 online supplemental file 2

10.1136/jech-2024-222654 online supplemental file 3

10.1136/jech-2024-222654 online supplemental file 4

10.1136/jech-2024-222654 online supplemental file 5

## Data Availability

Data sharing not applicable as no datasets generated and/or analysed for this study.
