# Supplementary material for: Minoritised ethnic groups and modifiable dementia risk: a scoping review of UK-based evidence
Source: J Epidemiol Community Health. 2025 Apr 17;79(9):e222654. doi: 10.1136/jech-2024-222654 (PMC12418548; doi:10.1136/jech-2024-222654)
Supplement: online supplemental file 2 [file jech-79-9-s002.docx]

**Supplement 2.** Search strategy in MEDLINE

Supplementary material for Jordão, M., Gong, L., Andre, D., Akhtar, A., Nwofe, E., Hawkins, R., Best, K., Parveen, S., Windle, K., & Clegg, A. Minoritised ethnic groups and modifiable dementia risk: a scoping review of UK-based evidence

Database: Ovid MEDLINE(R) ALL <1946 to May 18, 2023>

Search Strategy:

--------------------------------------------------------------------------------

1 Dementia/ (61241)

2 dementia.ti. (59745)

3 dementia.ab. /freq=2 (58068)

4 alzheimer disease/ (117400)

5 (alzheimer* or alzeimer*).ti. (87953)

6 (alzheimer* or alzeimer*).ab. /freq=2 (36072)

7 Cognitive Dysfunction/ (35032)

8 (cognit* adj2 (disorder* or dysfunction or impair* or decline or disparit* or deficit*)).ti. (44125)

9 (cognit* adj2 (disorder* or dysfunction or impair* or decline or disparit* or deficit*)).ab. /freq=2 (56511)

10 or/1-9 [dementia] (269200)

11 Minority Group/ (17746)

12 Minority Health/ (893)

13 ((racial or ethnic) adj1 minorit*).ti. (3107)

14 ((racial or ethnic) adj1 minorit*).ab. /freq=2 (4425)

15 Racial Groups/ (25418)

16 race factors/ (1136)

17 race/ (25418)

18 race.ti. (19693)

19 race.ab. /freq=2 (36054)

20 ethnic* group*.ti. (3772)

21 ethnic* group*.ab. /freq=2 (9125)

22 Population Group/ (5542)

23 ((racial or ethnic) adj5 disparit*).ti. (6564)

24 ((racial or ethnic) adj5 disparit*).ab. /freq=2 (3736)

25 people of colo?r.ti. (164)

26 people of colo?r.ab. /freq=2 (177)

27 Asian continental ancestry group/ (73399)

28 (BAME or BME).ti. (256)

29 (BAME or BME).ab. /freq=2 (1818)

30 exp "indigenous people"/ (36011)

31 indigenous.ti. (12497)

32 indigenous.ab. /freq=2 (11977)

33 "asian american"/ (9013)

34 migrant/ (14169)

35 "Emigrants and Immigrants"/ (15107)

36 Refugees/ (13093)

37 (aborig* or torres strait islander* or inuit* or eskimo* or kalaallit* or amerind* or romany or romanies or gypsies or gipsies or irish traveller* or first nation*).ti. (10529)

38 (aborig* or torres strait islander* or inuit* or eskimo* or kalaallit* or amerind* or romany or romanies or gypsies or gipsies or irish traveller* or first nation*).ab. /freq=2 (9569)

39 exp "Hispanic or Latino"/ (37932)

40 exp black people/ (96686)

41 (black or blacks or hispanic* or ((afro or african or asian or latin* or mexican or indian or native) adj1 american*)).ti. (87911)

42 (black or blacks or hispanic* or ((afro or african or asian or latin* or mexican or indian or native) adj1 american*)).ab. /freq=2 (125427)

43 south asian people/ (35)

44 south asian*.ti. (2831)

45 south asian*.ab. /freq=2 (3159)

46 Caribbean Region/ (5449)

47 Caribbean People/ (12)

48 Afr* Caribbean.ti. (558)

49 Afr* Caribbean.ab. /freq=2 (600)

50 (pacific island* or hawai*).ti. (7228)

51 (pacific island* or hawai*).ab. /freq=2 (5908)

52 Bangladesh/ (14271)

53 Bangladeshi/ (0)

54 bangladesh*.ti. (12562)

55 bangladesh*.ab. /freq=2 (8740)

56 Ukraine/ (17099)

57 Ukrain*.ab. /freq=2 (2494)

58 Ukrain*.ti. (4767)

59 White People/ (71398)

60 or/11-59 [minority groups] (501556)

61 (risk adj5 (dementia or alzheimer*)).tw. (20546)

62 ((vascular or cerebrovascular) adj risk).tw. (9549)

63 Educational status/ (58799)

64 education status.tw. (1118)

65 ((worse or less) adj educated).tw. (4580)

66 educational attainment.tw. (10896)

67 Hypertension/ (256814)

68 hypertens*.tw. (497343)

69 ((elevat*or high* or raised) adj2 (bp or blood pressure)).tw. (1665)

70 Deafness/ (28668)

71 exp Hearing Disorders/ (96621)

72 Persons With Hearing Impairments/ (3050)

73 (deaf* or "hear* impair*" or "hard of hear*" or "hard-of-hear" or "hear* difficult*" or "hear* disorder*" or hearing loss or hearing aid*).tw. (102179)

74 exp "tobacco use"/ (9471)

75 (smok* or tobacco* or cigarette*).tw. (395918)

76 drinking behavior/ (6884)

77 (alcohol* adj2 (drink* or consumption)).tw. (66732)

78 (drinking adj5 (behavio?r or habit*)).tw. (9448)

79 obesity/ (214503)

80 obesity.tw. (298980)

81 Depression/ (149636)

82 depressive disorder/ (74981)

83 (depressi* or depressed).tw. (533011)

84 Sedentary behavior/ (13493)

85 ((sedentary or sitting or seated) adj3 (behavio* or lifestyle or life-style or pattern* or leisure or time or bout* or prolonged)).tw. (19567)

86 physical* inactiv*.tw. (11969)

87 Diabetes mellitus/ (137729)

88 diabetes.tw. (633196)

89 social participation/ (3420)

90 social support/ (78546)

91 (social adj2 (integrat* or engag* or network* or ties)).tw. (36331)

92 (participat* adj2 (community or social or activit*)).tw. (23340)

93 interpersonal relations/ (76922)

94 (social adj5 alienat*).tw. (347)

95 (social adj5 isolat*).tw. (13958)

96 Social Alienation/ (1410)

97 Social Isolation/ (16142)

98 loneliness/ (6164)

99 Craniocerebral Trauma/ (23583)

100 Brain Edema/ (15452)

101 Cerebrovascular Trauma/ (273)

102 ((head or crani* or cerebr* or capitis or brain* or forebrain* or skull* or hemispher* or intra cran* or inter cran*) adj3 (injur* or trauma* or damag* or wound* or fracture* or contusion*)).tw. (178394)

103 ((brain or cerebral or intracranial) adj3 (oedema or edema or swell*)).tw. (19062)

104 Air Pollutants/ (57824)

105 Air Pollution/ (39516)

106 Environmental Exposure/ (82067)

107 exp Particulate Matter/ (78515)

108 Vehicle Emissions/ (11706)

109 Traffic Related Pollution/ (212)

110 (environment* adj3 (expos* or toxic* or contaminat*)).tw. (65021)

111 (particulate* adj3 (matter or air)).tw. (29050)

112 ((smog or fume* or exhaust* or diesel) and air).tw. (6887)

113 ((vehicle or traffic) adj3 (emission* or pollut*)).tw. (6168)

114 ((air or ambient) adj3 (pollut* or quality)).tw. (53067)

115 air quality/ (39516)

116 or/61-115 [risk factors] (3106584)

117 10 and 60 and 116 (2161)

118 (exp adolescent/ or exp child/ or exp newborn/) not ((exp adult/ or exp aged/ or exp middle aged/) and (exp adolescent/ or exp child/ or exp newborn/)) (1988120)

119 exp animals/ not humans.sh. (5122628)

120 letter/ (1217128)

121 editorial/ (649855)

122 news/ (218941)

123 exp historical article/ (409753)

124 Anecdotes as Topic/ (4747)

125 comment/ (1007855)

126 case reports/ (2335945)

127 (letter or comment*).ti. (188080)

128 or/118-127 [excluded study types] (11412648)

129 117 not 128 (2089)
