# Supplementary material for: Minoritised ethnic groups and modifiable dementia risk: a scoping review of UK-based evidence
Source: J Epidemiol Community Health. 2025 Apr 17;79(9):e222654. doi: 10.1136/jech-2024-222654 (PMC12418548; doi:10.1136/jech-2024-222654)
Supplement: online supplemental file 3 [file jech-79-9-s003.docx]

**Supplement 3. Data extraction form generated in Covidence**

Supplementary material for Jordão, M., Gong, L., Andre, D., Akhtar, A., Nwofe, E., Hawkins, R., Best, K., Parveen, S., Windle, K., & Clegg, A. Minoritised ethnic groups and modifiable dementia risk: a scoping review of UK-based evidence

**General information**

**Study ID**

*Please create study ID by using the surname of the first author, year of publication and the initials of the publication (e.g., journal)*

**First author surname**

**Year of publication**

**Title**

*Title of paper / abstract / report that data are extracted from*

**Type of publication**

1. Governmental report
2. Peer reviewed journal
3. NGO report
4. Thesis
5. Other

**Study funding sources**

*If this is not mentioned please write NM*

**Possible conflicts of interest for study authors**

*If this is not mentioned please write NM*

**Characteristics of included studies**

**General Methods**

**Aim of study**

*Please described the aim of the study*

**Study design**

1. Randomised controlled trial
2. Non-randomised experimental study
3. Cohort study
4. Cross sectional study
5. Case control study
6. Systematic review
7. Case series
8. Case report
9. Economic evaluation
10. Other

**Data source**

*Was the study based on primary or secondary data?*

1. Primary
2. Secondary
3. Both

**If secondary data was used, specify the database**

*If secondary data is used, please specify. If not, write NA (Not applicable)*

**When was the data collected?**

*If this is not mentioned please write NM*

**Study region**

*Where in the UK did this study took place?*

1. All 4 UK nations
2. Scotland
3. Wales
4. England
5. Northern Ireland
6. Not mentioned
7. Other

**Study specific location**

*If the study took place in a specific region (e.g., London, North of England), please specify. If not, please write NA (Not applicable)*

**Ethnicity measure**

*Please provide additional details about the ethnicity measure (e.g., which list of categories was used, other criteria used to define ethnicity, like country of birth, etc.)*

1. Self-report according to UK census categories
2. Self-report according to categories designed by researchers
3. Self-report without pre-listed categories
4. Attributed by researchers
5. Other

**Ethnicity measure specify**

*Quote details of the ethnic measure, e.g., were the categories of the census presented to the participants.*

**Dementia outcome measure**

1. Cognitive function test
2. Dementia diagnosis
3. Other

**Dementia outcome measure specify**

*Quote details of the dementia risk measure, e.g., cognitive function tests, or diagnostic criteria for dementia used. If more than one outcome measure related to dementia risk is used, please include all of those described.*

**Participants**

**Sample characteristics**

*Was the sample drawn from the general population or a specific subgroup (e.g., specific condition)?*

1. General population
2. Specific subgroup

**Sample subgroup**

*If the sample includes only a specific subgroup of people (e.g., specific condition) please specify which.*

*If the sample is drawn from the general population write NA*

**Inclusion criteria**

*If this is not mentioned please write NM*

**Exclusion criteria**

*If this is not mentioned please write NM*

**Age inclusion criteria**

**Recruitment methods**

1. Community organisations
2. NHS services
3. Existing cohort
4. Medical records
5. Non-medical records
6. Door to door
7. Media advertisement
8. Other

**Total number of participants**

*If this is not mentioned please write NM*

**Mean age of the participants**

*If this is not mentioned please write NM*

**Median age of participants**

*If this is not mentioned please write NM*

**SD age of the participants**

*If this is not mentioned please write NM*

**Age range of the participants**

*If this is not mentioned please write NM*

**Which ethnic minority groups have been studied?**

*Please select the options as described by the authors*

1. Black British
2. Asian British
3. Asian
4. Mixed
5. Black Caribbean/African-Caribbean
6. South Asian
7. East Asian
8. White Irish
9. Black African
10. African
11. African or Caribbean
12. Black
13. Unknown
14. Non-white
15. UK-born
16. Other

**Has a majority white/caucasian group been included?**

1. Yes
2. No

**Have ethnic groups been compared?**

1. Yes
2. No

**If ethnic groups have been compared, please specify which**

**Baseline Population Characteristics**

*Extract for included groups. If the study includes groups not listed, please add relevant category to the data extraction sheet.*

|  | **Black** | **South Asian** | **Caucasian / White** | **White Irish** | **Black African** | **Black Caribbean/African-Caribbean** | **Asian** | **Mixed** | **African or Caribbean** | **Other** | **Unknown** | **Non-White** | **UK-born** | **Other White** |
| --- | --- | --- | --- | --- | --- | --- | --- | --- | --- | --- | --- | --- | --- | --- |
| **Age range** |  |  |  |  |  |  |  |  |  |  |  |  |  |  |
| **Age mean** |  |  |  |  |  |  |  |  |  |  |  |  |  |  |
| **Age median** |  |  |  |  |  |  |  |  |  |  |  |  |  |  |
| **Age SD** |  |  |  |  |  |  |  |  |  |  |  |  |  |  |
| **Number of participants** |  |  |  |  |  |  |  |  |  |  |  |  |  |  |
| **Education level mean** |  |  |  |  |  |  |  |  |  |  |  |  |  |  |
| **Education level SD** |  |  |  |  |  |  |  |  |  |  |  |  |  |  |

**Analysis**

**MDF risk factors measured as predictors**

*Please select all that apply*

1. less education
2. hypertension
3. hearing impairment
4. smoking
5. obesity
6. depression
7. physical inactivity
8. diabetes
9. low social contact
10. alcohol consumption
11. traumatic brain injury
12. air pollution
13. none of the above

**MDF risk factors controlled for but not used as predictors**

*Please select all that apply*

1. less education
2. hypertension
3. hearing impairment
4. smoking
5. obesity
6. depression
7. physical inactivity
8. diabetes
9. low social contact
10. alcohol consumption
11. traumatic brain injury
12. air pollution
13. none of the above

**Other risk factors suggested by PPI**

*Were any of the following risk factors suggested by PPI included in the analysis (predictors or controlled variables)?*

1. sleep
2. diet
3. housing
4. sound pollution
5. low income
6. deprivation
7. racism/discrimination
8. visual impairment
9. smelling impairment
10. none of the above

**Risk factors measures**

*Please specify how each risk factor variable was measured if available (e.g., hypertension identified based in antihypertensive prescription)*

*Add Unspecified for risk factors which were analysed but don't mention how the risk factor was measured.*

|  | **Measured based on...** |
| --- | --- |
| **less education** |  |
| **hypertension** |  |
| **hearing impairment** |  |
| **smoking** |  |
| **obesity** |  |
| **depression** |  |
| **physical inactivity** |  |
| **diabetes** |  |
| **low social contact** |  |
| **alcohol consumption** |  |
| **traumatic brain injury** |  |
| **air pollution** |  |

**Statistical analysis/model**

*Please select the type of statistical analysis used*

1. Univariate
2. Multivariate
3. Both

**Interactions between risk factors**

1. Yes
2. No

**Interactions between risk factors and ethnicity**

1. Yes
2. No

**Main findings**

**Risk findings analysis adjustments**

*Do the authors report an adjusted and/or an unadjusted analysis of risk per ethnic group?*

1. Unadjusted
2. Adjusted
3. Both are presented
4. No risk findings analysis per ethnic group

**Risk findings adjusted variables and method**

*Please describe for which variables has the risk findings analysis been adjusted for, and the method used (NA, if not applicable)*

**Risk findings**

*Was significant risk found or not for risk factors and groups included in the analysis? Distinguish adjusted and unadjusted findings if available.*

*Extract for included groups. If the study includes groups not listed, please add relevant category to the data extraction sheet.*

|  | **Black** | **South Asian** | **Caucasian/White** | **White Irish** | **African** | **Black Caribbean/African-Caribbean** | **Asian** | **African or Caribbean** | **Other** | **Unknown** | **Non-white** | **UK-born** | **Other White** |
| --- | --- | --- | --- | --- | --- | --- | --- | --- | --- | --- | --- | --- | --- |
| **less education adjusted** |  |  |  |  |  |  |  |  |  |  |  |  |  |
| **hypertension adjusted** |  |  |  |  |  |  |  |  |  |  |  |  |  |
| **hearing impairment adjusted** |  |  |  |  |  |  |  |  |  |  |  |  |  |
| **smoking adjusted** |  |  |  |  |  |  |  |  |  |  |  |  |  |
| **obesity adjusted** |  |  |  |  |  |  |  |  |  |  |  |  |  |
| **depression adjusted** |  |  |  |  |  |  |  |  |  |  |  |  |  |
| **physical inactivity adjusted** |  |  |  |  |  |  |  |  |  |  |  |  |  |
| **diabetes adjusted** |  |  |  |  |  |  |  |  |  |  |  |  |  |
| **low social contact adjusted** |  |  |  |  |  |  |  |  |  |  |  |  |  |
| **alcohol consumption adjusted** |  |  |  |  |  |  |  |  |  |  |  |  |  |
| **traumatic brain injury adjusted** |  |  |  |  |  |  |  |  |  |  |  |  |  |
| **air pollution adjusted** |  |  |  |  |  |  |  |  |  |  |  |  |  |
| **less education unadjusted** |  |  |  |  |  |  |  |  |  |  |  |  |  |
| **hypertension unadjusted** |  |  |  |  |  |  |  |  |  |  |  |  |  |
| **hearing impairment unadjusted** |  |  |  |  |  |  |  |  |  |  |  |  |  |
| **smoking unadjusted** |  |  |  |  |  |  |  |  |  |  |  |  |  |
| **obesity unadjusted** |  |  |  |  |  |  |  |  |  |  |  |  |  |
| **depression unadjusted** |  |  |  |  |  |  |  |  |  |  |  |  |  |
| **physical activity unadjusted** |  |  |  |  |  |  |  |  |  |  |  |  |  |
| **diabetes unadjusted** |  |  |  |  |  |  |  |  |  |  |  |  |  |
| **low social contact unadjusted** |  |  |  |  |  |  |  |  |  |  |  |  |  |
| **alcohol consumption unadjusted** |  |  |  |  |  |  |  |  |  |  |  |  |  |
| **traumatic brain injury unadjusted** |  |  |  |  |  |  |  |  |  |  |  |  |  |
| **air pollution unadjusted** |  |  |  |  |  |  |  |  |  |  |  |  |  |

**Missing data risk findings**

*Was there missing data in the risk factors analysis?*

1. Yes
2. No
3. Not mentioned
4. No risk analysis per subgroup

**% Missing data risk findings**

*If there is missing data in the analysis, which % is this? If there is no missing data or no risk analysis per subgroup, please write NA*

**Imputation risk findings**

*If there is missing data in the analysis, was imputation used? If there is no missing data/not mentioned please chose NA (not applicable)*

1. Yes
2. No
3. No risk analysis per subgroup
4. NA

**Interactions between 12 MDF**

*Please describe the main findings on interactions between risk factors whenever available. Distinguish adjusted and unadjusted findings if available.*

|  | **Less education** | **Hypertension** | **Hearing impairment** | **Smoking** | **Obesity** | **Depression** | **Physical inactivity** | **Diabetes** | **Low social contact** | **Alcohol consumption** | **Traumatic brain injury** |
| --- | --- | --- | --- | --- | --- | --- | --- | --- | --- | --- | --- |
| **Less Education** |  |  |  |  |  |  |  |  |  |  |  |
| **Hypertension** |  |  |  |  |  |  |  |  |  |  |  |
| **Hearing impairment** |  |  |  |  |  |  |  |  |  |  |  |
| **Smoking** |  |  |  |  |  |  |  |  |  |  |  |
| **Obesity** |  |  |  |  |  |  |  |  |  |  |  |
| **Depression** |  |  |  |  |  |  |  |  |  |  |  |
| **Physical inactivity** |  |  |  |  |  |  |  |  |  |  |  |
| **Diabetes** |  |  |  |  |  |  |  |  |  |  |  |
| **Low social contact** |  |  |  |  |  |  |  |  |  |  |  |
| **Alcohol consumption** |  |  |  |  |  |  |  |  |  |  |  |
| **Traumatic brain injury** |  |  |  |  |  |  |  |  |  |  |  |
| **Air pollution** |  |  |  |  |  |  |  |  |  |  |  |

**Interactions between 12 MDF and other risk factors**

*If there is an analysis of interactions between the 12 MRF or the other risk factors mentioned above (sleep, diet, housing, sound pollution, low income, deprivation, racism/discrimination, visual impairment, smelling impairment), please specify.*

*If not, please write NA (Not applicable)*

**Risk factors x ethnicity adjustments**

*Do the authors report an adjusted and/or an unadjusted analysis of risk factors interaction with ethnicity?*

1. Unadjusted
2. Adjusted
3. Both are presented
4. No risk factors x ethnicity analysis

**Risk factor x ethnicity adjusted variables and method**

*Please describe for which variables has the risk factor x ethnicity analysis been adjusted for, and the method used (NA, if not applicable)*

**Interaction risk factors and ethnicity**

*If there is an analysis of interactions between risk factors and ethnicity please specify which and its main findings.*

*If not, please write NA (Not applicable)*

**Missing data risk factors x ethnicity**

*Was there missing data in the risk factors x ethnicity analysis?*

1. Yes
2. No
3. Not mentioned
4. No analysis of risk factors x ethnicity

**% Missing data risk factors x ethnicity**

*If there is missing data in the analysis, which % is this? If there is no missing data or no risk factors x ethnicity analysis, please write NA*

**Imputations risk factors x ethnicity**

*If there is missing data in the analysis, was imputation used? If there is no missing data/not mentioned please chose NA (not applicable)*

1. Yes
2. No
3. No analysis of risk factors x ethnicity
4. NA
