# Supplementary material for: Minoritised ethnic groups and modifiable dementia risk: a scoping review of UK-based evidence
Source: J Epidemiol Community Health. 2025 Apr 17;79(9):e222654. doi: 10.1136/jech-2024-222654 (PMC12418548; doi:10.1136/jech-2024-222654)
Supplement: online supplemental file 4 [file jech-79-9-s004.docx]

**Supplement 4.** Study characteristics, and details for ethnicity, modifiable risk factors and methods. Notes: *N includes all eligible participants. Participants lost to the analysis are detailed as missing data. 12 MRF: 12 modifiable risk factors for dementia.

Supplementary material for Jordão, M., Gong, L., Andre, D., Akhtar, A., Nwofe, E., Hawkins, R., Best, K., Parveen, S., Windle, K., & Clegg, A. Minoritised ethnic groups and modifiable dementia risk: a scoping review of UK-based evidence

| **Study ID** | **Study region** | **Study recruitment location** | | **Data collection date** | | **Aim** [quotes] | **Initial N*** | **Ethnicity categories** | **N* per ethnicity** | **Ethnicity definition** | |  |
| --- | --- | --- | --- | --- | --- | --- | --- | --- | --- | --- | --- | --- |
| Adelman 2009 (1) | England | London (Islington and South London) | | Detailed for included studies: Stewart et al 2001; Stewart et al 2003; Stevens et al 2004 | | To collate evidence regarding the prevalence and predictors of dementia or relative cognitive impairment in older, African-Caribbean people in Britain, as compared to their white, British peers. | 583 | African or Caribbean; black/African-Caribbean; UK-born | See included studies: Stewart et al 2001; Stewart et al 2003; Stevens et al 2004 | See included studies: Stewart et al 2001; Stewart et al 2003; Stevens et al 2004 | |  |
| Bature 2018 (2) | England | Milton Keynes and Luton | | 2006 to 2016 for dementia diagnosis; retrospective health data from 27 years before on average | | To identify patterns in signs and symptoms preceding the clinical diagnosis of AD to suggest a predictive model for earlier diagnosis of the disease in the primary care | 109 | Asian; black African; black/African-Caribbean; Middle Eastern; mixed; white | Non-White: 31  White: 78 | Based on medical records | |  |
| Bonnechere 2023 (3, 4) | England, Scotland and Wales | Edinburgh, Glasgow, Newcastle, Middlesbrough, Leeds, Sheffield, Bury, Manchester, Stockport, Liverpool, Wrexham, Stoke, Nottingham, Birmingham, Oxford, Reading, London, Croydon, Hounslow, Bristol, Cardiff, Swansea | | 2006 to 2010 | | To quantify risk factors of dementia, stroke, and mortality in Asian and black participants compared to whites. | 272660 | Asian; black;  white | Asian: 3686  Black: 2303  White: 266671 | Self-reported based on UK census categories | |  |
| Bothongo 2022 (5, 6) | England | East London: Hackney & City of London, Newham, Tower Hamlets, and Waltham Forest | | July 2009 to January 2018 | | To evaluate the relationships between ethnicity, area level socioeconomic deprivation and dementia risk | 19891 | Black; other; South Asian; unknown; white; | Black: 3769  White: 10301  Other: 1479  South Asian: 3232  Unknown: 1110 | Self-reported based on UK census categories | |  |
| Mukadam 2022 (7) | England, Scotland and Wales | Edinburgh, Glasgow, Newcastle, Middlesbrough, Leeds, Sheffield, Bury, Manchester, Stockport, Liverpool, Wrexham, Stoke, Nottingham, Birmingham, Oxford, Reading, London, Croydon, Hounslow, Bristol, Cardiff, Swansea | | Baseline: 2006 to 2010; Follow-up: continuously until 2020 | | To address a critical need to understand risks for all-cause dementia across ethnic groups, and whether ethnicity affects the association between established risk factors and dementia risk | 294162 | Black; South Asian; white; | Black: 2766  South Asian: 3590  White: 287806 | Self-reported based on UK census categories | |  |
| Mukadam 2023 (8-10) | England | Not applicable | | 1997 to 2018 | | To investigate ethnic differences in the associations of potentially modifiable risk factors with dementia | 1189090 | Black; other; South Asian; unknown; white | Black: 9166  Other: 13860  South Asian: 13082  Unknown: 322441  White: 830541 | Based on medical records | |  |
| Richards 2000 (11) | England | Bellenden Ward and London borough of Southwark | | Not mentioned | | To shed light on sources of bias in interpreting cognitive test scores obtained in cultures different from those on which they were standardised | 90 | Black/African-Caribbean; white | Black/African-Caribbean: 45  White: 45 | Self-reported based on UK census categories | |  |
| Sharma 2022 (12) | All 4 UK nations | Not applicable | | 1999 to 2018 | | To compare 20-year cardiometabolic trajectories, stratified by ethnicity, in those with type 2 diabetes who did and did not develop dementia | 117730 | Non-white; white | Not available | Based on medical records | |  |
| Stevens 2004 (13) | England | Islington, London | | Not mentioned | | To determine the association between African/Caribbean COB (country of birth) compared to UK COB and type of dementia; To explore whether people of African/Caribbean birth with hypertension were taking the treatments for this group which have been demonstrated to be the most effective | 1085 | African or Caribbean; UK-born | African or Caribbean: 98  UK-born: 667 | Self-reported country of birth | |  |
| Stewart 2001 (14) | England | South London | | Not mentioned | | To ascertain clinical vascular disease and risk in an older British African-Caribbean population and investigate associations with cognitive impairment | 290 | Black/African-Caribbean | Black/African-Caribbean: 290 | Attributed by clinical staff, subsequently confirmed based on participants self-reported country of birth and ancestry | |  |
| Stewart 2003 (15) | England | South London | | Baseline: 1997 to 1998; Follow-up: 2000 to 2001 | | To investigate associations between baseline factors and subsequent cognitive decline in an older African-Caribbean population | 216 | Black/African-Caribbean | Black/African-Caribbean: 216 | Attributed by clinical staff, subsequently confirmed based on participants self-reported country of birth and ancestry | |  |
| Stewart 2012 (16, 17) | England | Southall and Brent, London | | Baseline: 1988 to 1991; Follow-up: 2008-2011 | | To estimate the relationships between vascular risk factors and cognitive impairment among three ethnic groups (Black/South Asian/White UK residents) | 1187 | Black; South Asian; white | Black: 185  South Asian: 432  White: 570 | Attributed by researchers | |  |
| Taylor 2013 (18) | England | Southall and Brent, London | | Baseline: 1988 to 1991; Follow-up: 2008-2011 | | To investigate long-term prospective associations between a range of measurements of hypertensive status in midlife and cognitive impairment 20 years later | 4857 | Black/African-Caribbean; South Asian; white | Black/African-Caribbean: 241  South Asian: 551  White: 692 | Attributed by researchers | |  |
| Tsamakis 2021 (19, 20) | England | Southeast London | | 2007 to 2015 | | To compare ethnic group differences in symptom profile, functioning and pharmacotherapy at dementia diagnosis | 12154 | Black African; black/African-Caribbean; other white; South Asian; white Irish; white | Black African: 310  Black/African-Caribbean: 1661  Other White: 773  South Asian: 364  White Irish: 626 White: 8420 | Based on medical records | |  |
| **Study ID** | **12 MDF predictors** | | **12 MDF controlled** | | **Risk factors definitions** | | | | | | **Other risk factors** | |
| Adelman 2009 (1) | less education; hypertension; physical inactivity; diabetes | | none | | See included studies: Stewart et al 2001; Stewart et al 2003; Stevens et al 2004 | | | | | | none | |
| Bature 2018 (2) | hearing impairment; depression | | none | | Hearing impairment: based on medical records, otherwise unspecified  Depression: based on medical records, otherwise unspecified | | | | | | smelling impairment | |
| Bonnechere 2023 (3, 4) | less education; hypertension; hearing impairment; smoking; obesity; depression; physical inactivity; diabetes; low social contact; alcohol consumption; traumatic brain injury; air pollution | | none | | Education: low education when participants did not have a college or university degree, or A levels or equivalent (academic advanced-levels, post-compulsory education)  Hypertension: self-report of hypertension medication and/or mean of 2 blood pressure readings >=140 systolic blood pressure or >=90 diastolic blood pressure  Hearing impairment: self-report of hearing difficulties or deafness  Smoking: classified as never, previous or current presumably based on self-report  Obesity: >30 kg/m2 calculated from measured weight and height  Depression: self-report or record of depression episode (single episode, recurrent moderate, recurrent severe) by the first assessment  Physical inactivity: low IPAQ (International Physical Activity Questionnaire)  Diabetes: self-reported diabetes or record of any type of diabetes (type 1, 2, gestational and insipidus), or HbA1c >= 6.5% (48 mmol/mol)  Low social contact: no self-reported social group activity  Alcohol consumption: presumably based on self-report and categorised as: None - less than one dose per week; low - 1-2 per week; Mid - 3-4 per week; High - daily or almost daily  Traumatic brain injury: self -report or record of neurological / intracranial injury or trauma and fracture of skull or head before the first assessment  Air pollution: PM2.5, otherwise unspecified | | | | | | none | |
| Bothongo 2022 (5, 6) | hypertension; hearing impairment; smoking; obesity; depression; diabetes; alcohol consumption; traumatic brain injury | | none | | Hypertension: medical record diagnosis  Hearing impairment: medical record diagnosis or referral for hearing assessment  Smoking: categorised current, previous, or never having smoked, based on medical records.  Obesity: categories based on BMI calculated using height and weight measurements and participants were categorised as overweight/obese (BMI 25-50 kg/m2), normal weight (BMI 20- 24.9kg/m2), or underweight (10-19.9kg/m2).  Depression: medical record diagnosis  Diabetes: medical record diagnosis  Alcohol consumption: medical record of high alcohol intake  Traumatic brain injury: medical record diagnosis | | | | | | deprivation | |
| Mukadam 2022 (7) | less education; hypertension; hearing impairment; smoking; obesity; depression; physical inactivity; diabetes; low social contact; alcohol consumption; air pollution | | none | | Education: self-report education up to age 16 and higher than this (two categories)  Hypertension: self-report or reported use of hypertensive medications  Hearing impairment: self-reported problems with hearing including in a noisy environment; use of hearing aid  Smoking: self-report as current or non-current smokers  Obesity: BMI>30kg/m2 based on weight and height at baseline  Depression: self-report of having ever seen the doctor for anxiety or depression  Physical inactivity: self-reported duration, intensity and frequency, over previous four-week and  categorized as meeting the WHO guidance for physical activity or not (metabolic equivalent per week calculated based on published guidance)  Diabetes: self-report of diabetes diagnosis or the use of diabetes medication  Low social contact: cohabitation and self-reported frequency of contact with friends and family, used to categorise those with daily/almost daily social contact and less frequent contact  Alcohol consumption: self-report of frequency, quantity and type of alcohol per week. Categorised as drinking no alcohol, up to 21 or less units, or over 21 units per week splitting into concentrations below or equal to and above the WHO recommended threshold of an annual average  Air pollution: exposed to PM2.5 air pollution or not, based on estimates for the year 2010 modelled for each address as part of the European Study of Cohorts for Air Pollution Effects (ESCAPE). | | | | | | deprivation | |
| Mukadam 2023 (8-10) | hypertension; hearing impairment; smoking; obesity; depression; diabetes; alcohol consumption; traumatic brain injury | | none | | Hypertension: at least two of the following before age 65: hypertension diagnostic code in CPRD or HES, blood pressure reading of >=140mmHg systolic or >=90mmHg diastolic, and at least two prescriptions of antihypertensive medications.  Hearing impairment: sensorineural/central hearing loss or mixed hearing loss or presbycusis, not purely conductive hearing loss, at any age in medical record. Record of hearing aids (except bone-anchored). Referrals to audiology and testing. Congenital hearing loss, deafness as part of a medical syndrome, deaf-blind are excluded  Smoking: medical record of current smoker or non-smoker at the closest date of the start of the cohort  Obesity: any of the following before age 65: medical record of obesity, prescription of anti-obesity medications, referral for or record of bariatric surgery, recorded BMI of >=30kg/m2.  Depression: any record of depression or depressive symptoms  Diabetes: any of the following: medical record of diabetes or diabetes complications, prescription of diabetes medication, HbA1c >48 mmol/mol or 6.5%, plasma glucose >11.1 mmol/l, fasting plasma glucose>7.0 mmol/l, post-prandial glucose>11.1mmol/l, or v) abnormal glucose tolerance test (>11mmol/l after 2 hours).  Alcohol consumption: medical record of binge or excess alcohol consumption, or excess alcohol based on records indicating consumption of >=14 units of alcohol per week for women and >=21 units per week for men was classified as drinking excess alcohol  Traumatic brain injury: medical record of “concussion”, “cerebral contusion”, “head injury”, “brain injury”, “intracranial injury”, head or skull fracture, subarachnoid haemorrhage, extradural and  subdural haemorrhage related to injury. Intracerebral haemorrhage excluded if not caused by trauma. | | | | | | sleep; deprivation | |
| Richards 2000 (11) | None | | less education; hypertension; diabetes | | Education: years of education  Hypertension: presumably based on self-report of medical history  Diabetes: presumably based on self-report of medical history | | | | | | none | |
| Sharma 2022 (12) | hypertension; diabetes | | none | | Hypertension: mean systolic blood pressure  Diabetes: glycaemic control, fasting plasma glucose, HbA1c | | | | | | none | |
| Stevens 2004 (13) | hypertension | | smoking; diabetes; alcohol consumption | | Hypertension: blood pressure > 140/90 mm Hg. physical examination  Other MRF unspecified | | | | | | none | |
| Stewart 2001 (14) | less education; hypertension; smoking; obesity; physical inactivity; diabetes; alcohol consumption | | depression | | Education: low education was defined as leaving school with less than 15 years old, around 8 years of education. Leaving school at 15 or older was categorized as normal/high education.  Hypertension: diagnosis self-reported and medical examination including resting blood pressure, hypertension with proteinuria or hypertrophy on ECG  Smoking: structured questionnaire for smoking (Cox et al 1997, The Health and Lifestyle Survey)  Obesity: BMI and waist or waist/hip ratio  Depression: Geriatric Depression Scale (GDS) (10 item)  Physical inactivity: Structured questionnaire for physical activity - EPIC physical activity questionnaire (Pols et al., 1997)  Diabetes: previous diagnosis, diabetes with poor control defined as glycosuria or HbA1c >= 8.2.%  Alcohol consumption: Structured questionnaire for alcohol (Cox et al 1997, The Health and Lifestyle Survey) | | | | | | none | |
| Stewart 2003 (15) | less education; hypertension; physical inactivity; diabetes | | none | | Education: low education was defined as leaving school with less than 15 years old,  Hypertension: diagnosis self-reported and medical examination including resting blood pressure, hypertension with proteinuria or hypertrophy on ECG  Physical inactivity: Structured questionnaire for physical activity - EPIC physical activity questionnaire (Pols et al., 1997) A final item on the questionnaire asked whether participants had performed any regular activity over the last year that had caused them to sweat or increased their heart rate. A binary (present/absent) variable derived from this item was used a priori as a measure of activity.  Diabetes: Diabetes: previous diagnosis, diabetes with poor control defined as glycosuria or HbA1c >= 8.2.% Diabetes mellitus was categorized as severe if glycosuria was detected. | | | | | | none | |
| Stewart 2012 (16, 17) | hypertension; diabetes | | less education | | Education: years of education  Hypertension: resting blood pressure >140/90 or antihypertensive at treatment at baseline  Diabetes: unspecified | | | | | | none | |
| Taylor 2013 (18) | hypertension | | less education; smoking; obesity; diabetes; alcohol consumption | | Education: duration of education categorized as less than 10 years, or 10 years or more.  Hypertension: hypertension treatment, systolic and diastolic blood pressure, pulse pressure, mean arterial pressure, mean ambulatory SBP at 3-5am, 9-11am and 5-7pm, mean ambulatory DBP at 3-5am, 9-11am and 5-7pm.  Smoking: status was ascertained by questionnaire and coded as never, current, or previous  Obesity: BMI greater than 30.0 kg/m2  Diabetes: diabetes mellitus was ascertained from self-report and retrospective application of World Health Organization (WHO) 1999 criteria  Alcohol consumption: coded as less than weekly, one to two times per week, and daily or almost daily | | | | | | none | |
| Tsamakis 2021 (19, 20) | depression | | none | | Depression: depressive symptoms based on the Health of the Nation Outcome Scales for Elderly People (HoNOS 65+, Burns et al, 1999) | | | | | | housing; deprivation | |

| **Study ID** | **Study design** | **Data source and recruitment** | **Outcome measure** | **Type of analysis** | **Risk findings per subgroup** | **Interaction between 12 MRF and ethnicity** | **Interaction between risk factors** | **Adjustments for all performed analyses** | **Missing data** | **Imputation** |
| --- | --- | --- | --- | --- | --- | --- | --- | --- | --- | --- |
| Adelman 2009 (1) | Systematic review | Secondary, systematic search | See included studies: Stewart et al 2001; Stewart et al 2003; Stevens et al 2004 | See included studies | See included studies | See included studies | See included studies | See included studies | See included studies | See included studies |
| Bature 2018 (2) | Case control study | Secondary, Medical records of 3 GP practices | Dementia diagnosis based on medical record | Multivariate | No | Yes | No | Unadjusted | Not mentioned | Not applicable |
| Bonnechere 2023 (3, 4) | Cohort study | Primary; invitation sent based on population-based registers; UK Biobank | Dementia diagnosis based on self-report or medical record | Multivariate | Yes | Yes | No | Adjusted | Not mentioned | Not applicable |
| Bothongo 2022 (5, 6) | Case control study | Secondary; medical records for the Secure Health Analysis and Research in East London project | Dementia diagnosis based on medical record | Multivariate | No | Yes | Yes | Adjusted | <10% missing for any variable | Missing data for ethnicity was included in the analysis as unknown. Sensitivity analysis with imputation were conducted. |
| Mukadam 2022 (7) | Cohort study | Secondary; records in UK Biobank | Dementia diagnosis based on self-report or medical record | Multivariate | Yes | Yes | No | For subgroup analysis, adjusted and unadjusted analyses are presented. For Interaction between risk factors and ethnicity the analysis is adjusted | No missing data for: hypertension, hearing loss, obesity, physical inactivity and alcohol intake.  Missing data for: smoking (0.59%), deprivation (Townsend score, 0.13%), social isolation (0.20%), diabetes (0.52%), educational attainment (0.74%), ethnicity (0.74%), depressive symptoms (1.0%), and air pollution (8.22%). | Not mentioned |
| Mukadam 2023 (8-10) | Cohort study | Secondary; medical records in CALIBER (electronic patient health records from CPRD, linked to Hospital Episode Statistics and Mortality Statistics) | Dementia diagnosis based on medical record | Multivariate | Yes | Yes | No | Adjusted | 27% missing for ethnicity | In some subgroup analyses and in the interaction between risk factors and ethnicity, imputation was used. |
| Richards 2000 (11) | Cross sectional study | Primary; non-medical record list subsequent door to door contact | Dementia diagnosis based on cognitive and clinical assessment | Multivariate | Yes | No | No | Adjusted | 4.4% missing, partial data from direct and informant data in some cases | Not mentioned |
| Sharma 2022 (12) | Case control study | Secondary; medical records in the Clinical Practice Research Database | Dementia diagnosis based on medical record | Multivariate | Yes | No | No | Adjusted | Not mentioned | Not applicable |
| Stevens 2004 (13) | Cross sectional study | Primary; door to door | Dementia diagnosis based on cognitive and clinical assessment | Univariate | Yes | No | No | Unadjusted | Not mentioned | Not applicable |
| Stewart 2001 (14) | Cross sectional study | Primary; medical records and subsequent contact by NHS services | Cognitive function testing (cutoff not validated externally) | Uni and multivariate | Yes | No | Yes | Adjusted and unadjusted | 4% missing data due to lack of cognitive data; 16% missing data in the adjusted analysis | Not mentioned |
| Stewart 2003 (15) | Cohort study | Primary; medical records and subsequent contact by NHS services | Cognitive function testing (cutoff not validated externally) | Univariate | Yes | No | No | Unadjusted | 4.2% missing data | Not mentioned |
| Stewart 2012 (16, 17) | Cohort study | Primary; NHS services, primary care and industrial workforce lists | Cognitive function testing (cutoff not validated externally) | Multivariate | Yes | No | No | Adjusted | Not mentioned | Not applicable |
| Taylor 2013 (18) | Cohort study | Primary; traced participants in previous study through health service tracing system | Cognitive function testing (cutoff not validated externally) | Multivariate | No | Yes | No | Adjusted | 62% missing at 20 years follow up, and 7% additionally missing due to lack of cognitive data | Not mentioned |
| Tsamakis 2021 (19, 20) | Cross sectional study | Secondary; medical records in Clinical Record Interactive Search System | Dementia diagnosis based on medical record | Multivariate | No | Yes | No | Adjusted | 23% missing data on at least 1 covariate | Yes |

**References:**

1. Adelman S, Blanchard M, Livingston G, Adelman S, Blanchard M, Livingston G. A systematic review of the prevalence and covariates of dementia or relative cognitive impairment in the older African-Caribbean population in Britain. International Journal of Geriatric Psychiatry. 2009;24(7):657-65.

2. Bature F, Pang D, Robinson A, Polson N, Pappas Y, Guinn B. Identifying patterns in signs and symptoms preceding the clinical diagnosis of Alzheimer's disease: Retrospective medical record review study and a nested case-control design. Current Alzheimer Research. 2018;15(8):723-30.

3. Bonnechere B, Liu J, Thompson A, Amin N, van Duijn C. Does ethnicity influence dementia, stroke and mortality risk? Evidence from the UK Biobank. Frontiers in public health. 2023;11:1111321.

4. Biobank UK. Protocol for a large-scale prospective epidemiological resource2006. Available from: <https://www.ukbiobank.ac.uk/media/gnkeyh2q/study-rationale.pdf>.

5. Bothongo PLK, Jitlal M, Parry E, Waters S, Foote IF, Watson CJ, et al. Dementia risk in a diverse population: A single-region nested case-control study in the East End of London. The Lancet regional health Europe. 2022;15:100321.

6. Ethnicity and deprivation could link to dementia risk 2022 [Available from: <https://www.bartscharity.org.uk/our-news/ethnicity-and-deprivation-associated-with-dementia-risk/>.

7. Mukadam N, Marston L, Lewis G, Livingston G. Risk factors, ethnicity and dementia: A UK Biobank prospective cohort study of White, South Asian and Black participants. PLOS ONE. 2022;17(10):e0275309.

8. Mukadam N, Marston L, Lewis G, Mathur R, Lowther E, Rait G, Livingston G. South Asian, Black and White ethnicity and the effect of potentially modifiable risk factors for dementia: A study in English electronic health records. PLOS ONE. 2023;18(10):e0289893.

9. Brogan J. UCL study finds certain ethnicities experience greater effect of dementia risk factors: Researchers analysed health data from nearly one million adults in England 2023 [Available from: <https://www.pmlive.com/pharma_news/ucl_study_finds_certain_ethnicities_experience_greater_effect_of_dementia_risk_factors_1502428>.

10. Ethnic Minorities Experience Greater Effect Of Dementia Risk Factor, Study Suggests: The Carer; 2023 [Available from: <https://thecareruk.com/ethnic-minorities-experience-grater-effect-of-dementia-risk-factor-study-suggests/>.

11. Richards M, Brayne C, Dening T, Abas M, Carter J, Price M, et al. Cognitive function in UK community-dwelling African Caribbean and White elders: A pilot study. International Journal of Geriatric Psychiatry. 2000;15(7):621-30.

12. Sharma A, Lai H, Chang K, Sharabiani M, Bottle A, Valabhji J, et al. A 20-year follow-up of cardiometabolic trajectories amongst individuals with type 2 diabetes before dementia diagnosis by ethnic group. Diabetic Medicine. 2022;39(SUPPL 1):28.

13. Stevens T, Leavey G, Livingston G. Dementia and hypertension in African/Caribbean elders. Age & Ageing. 2004;33(2):193-5.

14. Stewart R, Richards M, Brayne C, Mann A. Vascular risk and cognitive impairment in an older, British, African-Caribbean population. Journal of the American Geriatrics Society. 2001;49(3):263-9.

15. Stewart R, Prince M, Mann A. Age, Vascular Risk, and Cognitive Decline in an Older, British, African-Caribbean Population. Journal of the American Geriatrics Society. 2003;51(11):1547-53.

16. Stewart R, Tillin T, Chaturvedi N. Vascular risk profiles and cognitive impairment in a 20-year follow-up of three ethnic groups: The southall and brent revisited (sabre) cohort. Stroke. 2012;43(2 Meeting Abstracts).

17. Tillin T, Forouhi NG, McKeigue PM, Chaturvedi N, for the SSG. Southall And Brent REvisited: Cohort profile of SABRE, a UK population-based comparison of cardiovascular disease and diabetes in people of European, Indian Asian and African Caribbean origins. International Journal of Epidemiology. 2012;41(1):33-42.

18. Taylor C, Tillin T, Chaturvedi N, Dewey M, Ferri CP, Hughes A, et al. Midlife hypertensive status and cognitive function 20 years later: The Southall and Brent Revisited Study. Journal of the American Geriatrics Society. 2013;61(9):1489-98.

19. Tsamakis K, Gadelrab R, Wilson M, Bonnici-Mallia AM, Hussain L, Perera G, et al. Dementia in People from Ethnic Minority Backgrounds: Disability, Functioning, and Pharmacotherapy at the Time of Diagnosis. Journal of the American Medical Directors Association. 2021;22(2):446-52.

20. People with dementia from ethnic minority backgrounds face extra barriers in accessing care: NIHR Alerts; [Available from: <https://evidence.nihr.ac.uk/alert/ethnic-minority-dementia-extra-barriers-in-accessing-care/>.
