# Supplementary material for: Minoritised ethnic groups and modifiable dementia risk: a scoping review of UK-based evidence
Source: J Epidemiol Community Health. 2025 Apr 17;79(9):e222654. doi: 10.1136/jech-2024-222654 (PMC12418548; doi:10.1136/jech-2024-222654)
Supplement: online supplemental file 5 [file jech-79-9-s005.docx]

|  | **African or Caribbean** | **Asian** | **Black** | **Black African** | **Black/African-Caribbean** | **Middle Eastern** | **Mixed** | **Non-white** | **Other** | **Other white** | **South Asian** | **UK-born** | **Unknown** | **White Irish** |
| --- | --- | --- | --- | --- | --- | --- | --- | --- | --- | --- | --- | --- | --- | --- |
| **Less education** |  | ● | ●●🞫 |  | ●●🞫🞫 |  |  |  |  |  | ●🞫🞫 |  |  |  |
| **Hypertension** | ● | ● | ●●●●🞫 |  | ●●●🞫 |  |  | 🞫 | 🞫🞫 |  | ●●●🞫🞫 | 🞫 | 🞫🞫 |  |
| **Hearing impairment** |  | ●🞫 | ●●🞫🞫 | 🞫 | 🞫 | 🞫 | 🞫 |  | 🞫🞫 |  | ●🞫🞫 |  | 🞫🞫 |  |
| **Smoking** | 🞫 | ● | ●●🞫🞫 |  | ●🞫 |  |  |  | 🞫🞫 |  | ●🞫🞫🞫 | 🞫 | 🞫🞫 |  |
| **Obesity** |  | ● | ●●●🞫 |  | ●🞫 |  |  |  | 🞫🞫 |  | ●●🞫🞫 |  | 🞫🞫 |  |
| **Depression** |  | ●🞫 | ●●🞫🞫 | 🞫🞫 | ●🞫🞫 | 🞫 | 🞫 |  | 🞫🞫 | 🞫 | ●🞫🞫🞫 |  | 🞫🞫 | 🞫 |
| **Physical inactivity** |  | ● | ●● |  | ●● |  |  |  |  |  | ● |  |  |  |
| **Diabetes** | 🞫 | ● | ●●●●🞫 |  | ●●●🞫 |  |  | ● | 🞫🞫 |  | ●●●🞫🞫 | 🞫 | 🞫🞫 |  |
| **Low social contact** |  | ● | ●● |  |  |  |  |  |  |  | ● |  |  |  |
| **Alcohol consumption** | 🞫 | ● | ●●🞫🞫 |  | ●🞫 |  |  |  | 🞫🞫 |  | ●🞫🞫🞫 | 🞫 | 🞫🞫 |  |
| **Traumatic brain injury** |  | 🞫 | ●🞫🞫 |  |  |  |  |  | 🞫🞫 |  | 🞫🞫 |  | 🞫🞫 |  |
| **Air pollution** |  | ● | ●● |  |  |  |  |  |  |  | ● |  |  |  |

Supplementary material for Jordão, M., Gong, L., Andre, D., Akhtar, A., Nwofe, E., Hawkins, R., Best, K., Parveen, S., Windle, K., & Clegg, A. Minoritised ethnic groups and modifiable dementia risk: a scoping review of UK-based evidence

**Supplement 5.** Representation of 12 MRF and minoritised ethnic groups in the included studies. Ethnic labels as in included studies. For ● findings for risk factors by subgroup are reported; for 🞫 subgroups and/or risk factors are included in the sample/data, but no subgroup findings are reported.
